# Resting state networks of the canine brain under sevoflurane anaesthesia

**DOI:** 10.1371/journal.pone.0231955

**Published:** 2020-04-17

**Authors:** Katrin M. Beckmann, Adriano Wang-Leandro, Matthias Dennler, Ines Carrera, Henning Richter, Rima N. Bektas, Aline Steiner, Sven Haller

**Affiliations:** 1 Neurology Department, Clinic of Small Animal Surgery, Vetsuisse Faculty Zurich, Zurich, Switzerland; 2 Department of Diagnostics and Clinical Services, Clinic for Diagnostic Imaging, Vetsuisse-Faculty Zurich, Zurich, Switzerland; 3 Willows Veterinary Centre and Referral Service, Shirley, United Kingdom; 4 Department of Diagnostics and Clinical Services, Section of Anaesthesiology, Vetsuisse Faculty, University of Zurich, Zurich, Switzerland; 5 Department of Surgical Sciences, Radiology, Uppsala University, Uppsala, Sweden; 6 Faculty of Medicine of the University of Geneva, Geneva, Switzerland; McLean Hospital, UNITED STATES

## Abstract

Resting-state functional Magnetic Resonance Imaging (rs-fMRI) has become an established technique in humans and reliably determines several resting state networks (RSNs) simultaneously. Limited data exist about RSN in dogs. The aim of this study was to investigate the RSNs in 10 healthy beagle dogs using a 3 tesla MRI scanner and subsequently perform group-level independent component analysis (ICA) to identify functionally connected brain networks. Rs-fMRI sequences were performed under steady state sevoflurane inhalation anaesthesia. Anaesthetic depth was titrated to the minimum level needed for immobilisation and mechanical ventilation of the patient. This required a sevoflurane MAC between 0.8 to 1.2. Group-level ICA dimensionality of 20 components revealed distributed sensory, motor and higher-order networks in the dogs’ brain. We identified in total 7 RSNs (default mode, primary and higher order visual, auditory, two putative motor-somatosensory and one putative somatosensory), which are common to other mammals including humans. Identified RSN are remarkably similar to those identified in awake dogs. This study proves the feasibility of rs-fMRI in anesthetized dogs and describes several RSNs, which may set the basis for investigating pathophysiological characteristics of various canine brain diseases.

## Introduction

Spontaneous fluctuations in activity in different parts of the brain can be used to study functional brain networks [1]. Increased neuronal activity leads to increased energy consumption. This energy is produced locally from glucose and oxygen supplied by blood vessels, leading to an increased oxygen consumption [2]. Blood oxygenation level-dependent (BOLD) functional magnetic resonance imagining (fMRI) technique takes advantage of the fact that oxyhaemoglobin is diamagnetic, and de-oxyhaemoglobin is paramagnetic which leads to an increased signal intensity in activated brain areas [3]. Resting-state fMRI (rs-fMRI) is based on spontaneous low frequency fluctuations (0.1 Hz) in the BOLD signal when the brain is at rest (not performing any specific task). Studying correlations between variations of the BOLD signal can identify anatomically distinct regions, which activate synchronously with each other, these regions are called “resting state networks” [4]. Resting state networks (RSN) have been identified in many species including rodents [5–7], ferrets [8], monkeys [9, 10] and humans [11, 12] and have been extensively used in cognitive neuroscience research [13].

BOLD task-fMRI has been used successfully to compare dogs’ and humans’ brain function under physiological [14] and pathological [15] conditions, but little is known about the rs-fMRI in dogs. To date two studies have been performed in healthy dogs. The first study identified one component of the RSN, the default mode network (DMN), in awake and anaesthetized dogs [16]. A very recent study found multiple, spatially distributed RSNs in awake dogs [17].

Increasing evidence from clinical rs-fMRI studies in humans has indicated that rs-fMRI may be a promising tool for investigating pathophysiological characteristics of RSNs associated with epilepsy and neurodegenerative diseases. Alterations in RSNs, correlating with the progression and severity of the diseases, can be found in the absence of structural lesions indicating the sensitivity of this method [18]. The role of the dog as an established large animal translational model has been increasingly recognized in multiple research fields of neuroscience including mental disorders [19, 20], aging [21] and naturally occurring neurological diseases such as epilepsy [22, 23]. Many questions are open regarding similarities and differences between these diseases in humans and dogs. Rs-fMRI, as a non-invasive method would allow investigation of alteration in the brain function of aging dogs or dogs suffering from epilepsy. It could be used to measure natural disease progression or response to new therapeutics, but it is necessary to collect a sufficient number of dogs to reflect variation within the population and to enable generalization of specific results from the examined dog. Previously published fMRI data from dogs are collected from small number of dogs. This is mainly because of the huge effort to train the dog for awake fMRI in order to get high quality data [19]. Idiopathic epilepsy for example is one of the most common chronic neurological diseases in dogs with an estimated prevalence of 0.6–0.75% in the general dog population [24, 25]. To take advantage of this considerably high number of dogs suffering from these diseases it is essential to find a more feasible approach than months of training for rs-fMRI. General anaesthesia is such an approach, especially because companion animals are routinely examined under anaesthesia for diagnostic purposes. From an animal welfare point of view this means data could be collected with minimal additional physical strain for the animals, but this requires the ability to reliably identify RSNs under routine general anaesthesia. It is well known that general aesthesia has an effect on detection of RSN [26–29]. And also, in human medicine awake rs-fMRI is preferred method, it has been suggested, that for those patients that cannot be scanned awake, rs-fMRI under general anaesthesia can add significant information [30–32]. For further investigations in dogs it is necessary to prove that RSNs can be detected under common aesthetic protocols in dogs.

The aim of this study was to investigate the RSNs in dogs using a standardized anaesthetic protocol. We hypothesize that not only the DMN, but also other higher order networks will be identified.

## Material and methods

### Animals

A total of 10 adult purpose-bred research Beagles were included in the study. Four dogs were females and six were males. All dogs were sexually intact. Age ranged from 2–6 years (mean age 4.8 years). The bodyweight was between 9.6 and 18.6 kg (mean weight 15.22 kg). For inclusion, clinical and neurological examination as well as blood biochemical analysis and haematology had to be within reference limits. The study was approved by the Cantonal Veterinary Office of Zurich (animal license number ZH272/16).

### Animal preparation

Dogs were pre-medicated with butorphanol (0.1–0.2mg/kg IM, depending on the temperament of the dog) prior to catheter placement. An IV catheter was placed in the cephalic vein using aseptic technique. Anaesthesia was induced with Propofol 1% (MCT Fresenius Kabi, Oberdorf, Switzerland) IV, given to effect. All dogs were oro-tracheally intubated following induction of anaesthesia. Anaesthesia was maintained with sevoflurane vaporized in oxygen and medical air. Vaporizer settings were adjusted depending on the depth of anaesthesia (0.8–1.2 mean alveolar concentration (MAC)). End tidal concentration of sevoflurane, heart rate (HR), respiratory rate (RR), non-invasive mean blood pressure, percutaneous arterial oxygen saturation (SpO2) and end tidal partial pressure of carbon dioxide (EtCO2) were monitored using medical monitor (DatexOhmeda) and recorded every 5 minutes. Normocapnia (EtCO2 between 35 and 38 mmHg) was maintained using a ventilator (tidal volume 10-15ml/kg). FiO2 was kept between 55–65%. Blood pressures were maintained stable within physiological values under general anaesthesia (Mean arterial blood pressure (MAP) > 60mmHg) using RiAc infusion.

MAP under 60mmHg was treated first with fluid/RiAc challenges of 3ml/kg IV administered over 5–10 minutes. After 3 fluid challenges of RiAc, a dobutamine constant rate infusion (1-5mcg/kg/min) was started to maintain MAP over 60mmHg.

### fMRI protocol

All MRI data were acquired with a 3 Tesla scanner (Philips Ingenia, Philips AG, Zurich, Switzerland) using a 16‐channel receive‐transmit head coil (dStream HeadSpine coil solution, Philips AG). For anatomical evaluation, a 3D T1-weighted (T1W; TR 13 ms; TE 6 ms; FOV 130 mm; slice thickness 0.6 mm; flip angle 8°) sequence was acquired. After the anatomical scans were obtained, around 1 h after induction of anaesthesia, rs-fMRI scans were acquired in all dogs.

BOLD functional resting state scans were acquired with gradient-echo planar imaging (EPI) sequence using the following protocol: TR = 2.0 s; TE = 30 ms; field of view (FOV) = 236 mm; slice thickness = 3 mm; acquisition time of 12.07 minutes.

### MR data pre-processing

DICOM images were converted to 4D NIFTI formatted-images using an open-source conversion software (dcm2nii, University of South Carolina, South Carolina, U.S). Further pre-processing was performed using the open source software FSL (FMRIB Software Library v6.0, Oxford, UK).

Orientation of the images was set according to neurological convention. T1W images were cropped in order to remove as much of the extra-calvarial structures such as skin and muscles, taking care of including all parts of the brain. In the next step the brain was extracted using automated method the brain extraction tool of FSL-BET [33] with parameters set at -f 0.15 and -g 0.

A study specific T1-template was created by registering all T1W images to the T1W image of one of the study dogs. In a second step the output images were averaged together using the FLIRT and fslmaths of the above mentioned software [34, 35] (S1 Fig).

**Fig 1:**
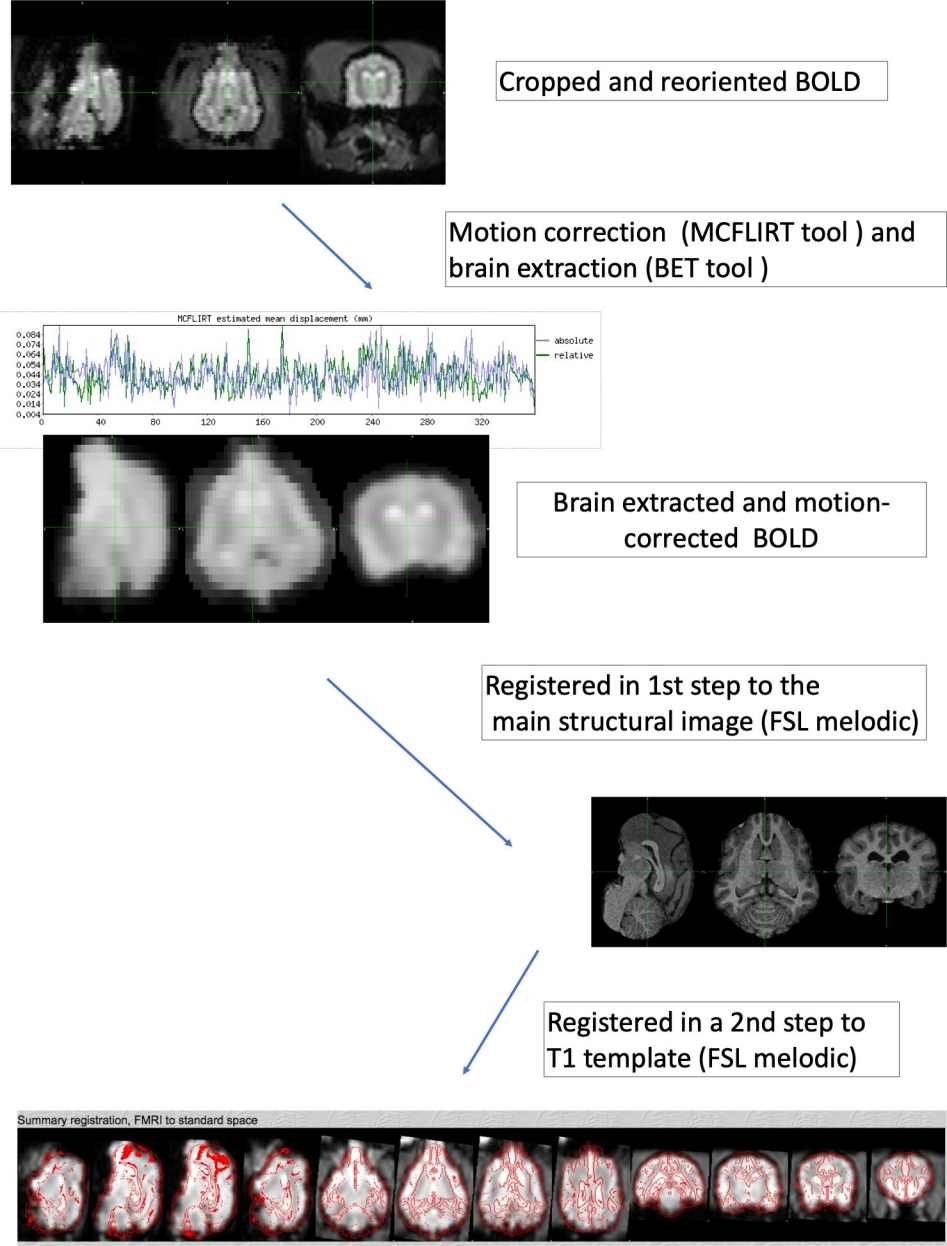
Schematic representation of the pre-processing of fMRI data. The first two steps (converting DICOM to NIFTI-files, reorienting and cropping are identical to the pre-processing of the anatomical images and not shown in this figure).

For correction of motion artefacts and high pass temporal filtering with sigma = 50.0 s, fMRI data underwent intra-modal motion correction (MCFLIRT [34]): the time series are in its entirety loaded and the middle volume represents the initial template image. A course 8-mm search for the motion parameters is then carried out using the cost function specified followed by two subsequent searches at 4 mm using increasingly tighter tolerances. All optimisations use tri-linear interpolation. In the second phase, an identity transformation is assumed between the middle volume and the adjacent volume. The transformation found in this first search is then used as the estimate for the transformation between the middle volume and the volume beyond the adjacent one [34]. Full width at half-maximum (FWHM) spatial smoothing was applied at the default 5mm [34]. FSL-BET tool was applied for brain extraction with parameters set at -f 0.35 and -g 0. Afterwards, registration of individual fMRI data was performed to the matching main structural image of this dog (3D-T1W sequence) and to a standard space (anatomical T1W template) using a linear approach with 12 degrees of freedom. The resampling resolution was set at 2mm (Fig 1).

### Independent component analysis

RSNs were generated by means of a multivariate data-driven ICA approach. As data quality control, different output independent components were initially assessed: 17, 20, 25 and 30. Individual ICs were visually compared and 20 components were selected by consensus of three authors (K.B, A.W-L, and S.H).

Criteria for selection of RSNs in the different components were the following: consisted of relatively large continuous regions of increased BOLD signal, 2) were largely bilateral and/or 3) could be referred to anatomical landmarks comparable to well-known structures in existing literature of dogs [14–16, 36–39].

For identification of brain regions within gICA connectivity maps were thresholded with red-yellow color encoding using a 3.5< Z-score, main foci were defined as regions that exhibited high BOLD signal (labelled in yellow) and satisfied the above criteria.

For statistical analysis, one sample t-test after dual regression analysis of the data was performed. Specifically, a set of spatial maps from the group-average analysis was used to generate subject-specific versions of the spatial maps, and associated time series, using dual regression [40]. First, for each subject, the group-average set of spatial maps is regressed (as spatial regressors in a multiple regression) into the subject's 4D space-time dataset. This results in a set of subject-specific time series, one per group-level spatial map. Afterwards, those time series are regressed (as temporal regressors, again in a multiple regression) into the same 4D dataset, resulting in a set of subject-specific spatial maps, one per group-level spatial map. Finally, one sample t-test was performed using FSL's randomize permutation-testing tool [41]. Clusters of increased BOLD signal within the RSNs were confirmed using a significance level of p<0.05.

## Results

### Image acquisition and pre-processing

Anaesthesia was uneventful in all dogs. Mean deviation time series of six affine parameters (three translations and three rotations) were plotted to check whether there was significant head movement during the functional scan.

HR varied during rs-fMRI between 78-150/min (median HR: 98/min; mean HR: 105/min. RR varied during rs-fMRI between 10-21/min (median RR: 15/min, mean RR: 15/min)

The motion was minimal in all dogs during the scan with a mean relative displacement of 0.05 mm (0.04–0.08mm) and a mean absolute displacement of 0.06 mm (0.03–0.09 mm; Fig 1).

### Identification of RSN

We used ICA to derive RSNs in the dogs’ brain. The components or putative networks were visually inspected and those not meeting the established inclusion criteria (n = 12) were excluded and the remaining components (n = 8) were further evaluated. Excluded components are shown in the S2 Fig. Excluded components were either localised completely (#1 & #2) or partially outside the brain (#3) or were clearly not overlaying grey matter (#4–12) and therefore originate most likely from non-neural physiological fluctuation [12].

**Fig 2:**
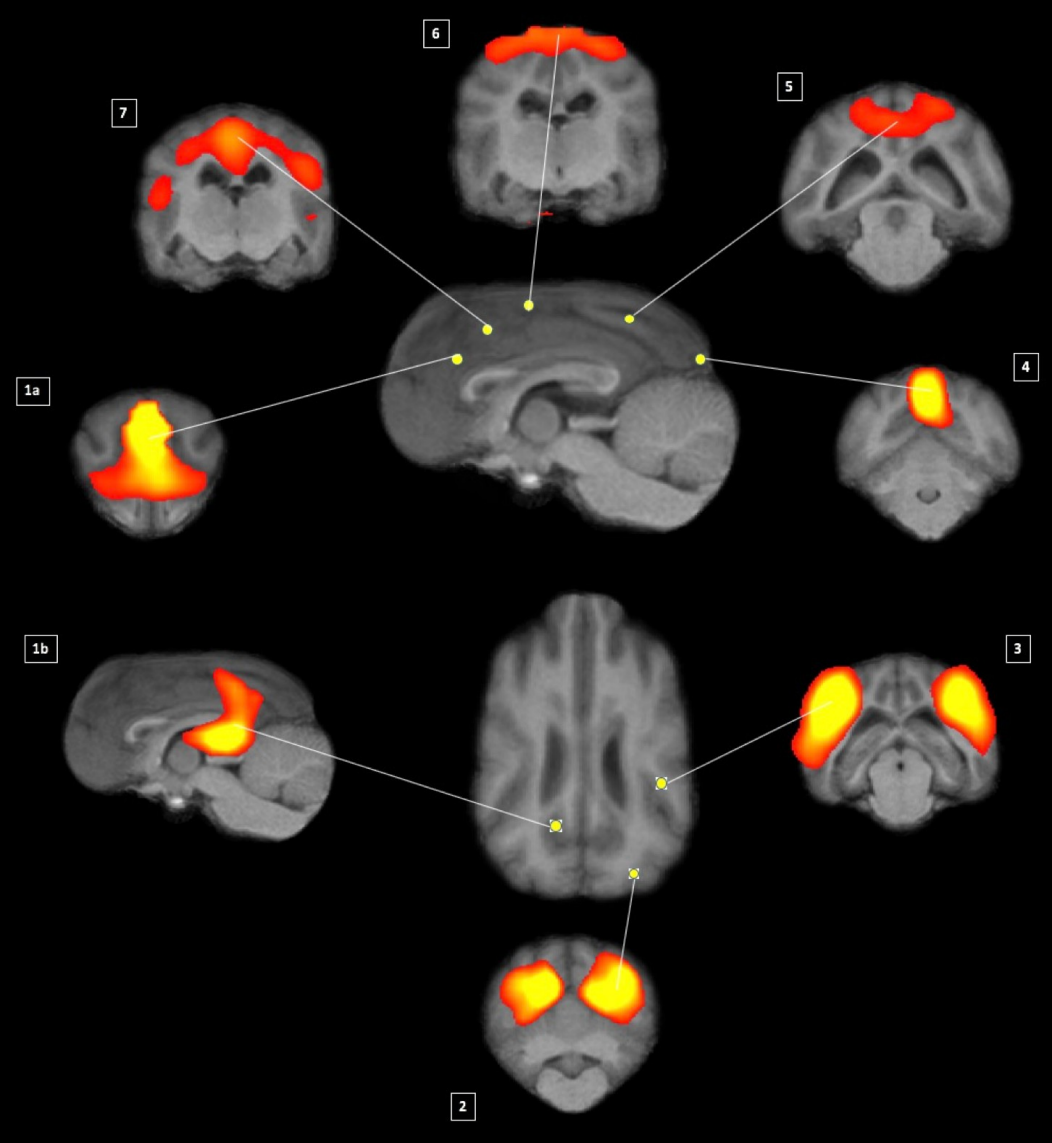
Maps of healthy beagle dog obtained by means of group independent component analysis and registered on T1 group-specific template with red-yellow color encoding using a 3.5< Z-score threshold: 1a and 2–7 transverse and 1b sagittal. Centre of the networks shown in the sagittal and dorsal view in the middle.

The remaining components were topological consistent with 7 RSNs observed in other species and included auditory(3), primary visual(4) higher order visual(2), DMN; 1a [anterior] & 1b [posterior], motor/somatosensory(6 and 7), and somatosensory(5) (Fig 2).

1a anterior DMN 1b posterior DMN, 2 higher order visual, 3 auditory, 4 primary visual, 5 somatosensory, 6 & 7 sensory-motor.

The main foci of each of functional (bolded) and anatomical structures are listed as follows:

We labelled the network 1a as **anterior DMN** as it was composed of main foci in medial prefrontal cortex. Additionally, network 1b was deemed **posterior DMN** as it composed of main foci in posterior parietal cortex and posterior cingulate cortex.

Main connectivity foci of the **auditory (network 3)** included peri-sylvian regions, including the Sylvian gyri along the Sylvian and the ecto-sylvian gyri along the ecto-sylvian sulcus and extending dorsally to the supra-sylvian sulcus.

The **primary visual (network 4)** included the primary visual cortex [42]: posterior, midline structure adjacent to the inter-hemispheric fissure. A second, **higher order visual (network 2)** was identified within the more laterally located in the caudal ecto-marginal and supra-sylvian areas.

Three further networks were identified in the parietal cortex: one putative **motor/somatosensory (network 7)** includes the pre- and post-cruciate gyrus, the rostral supra-sylvian gyrus, the rostral endo-marginal gyrus, rostral marginal, the rostral ecto-sylvian gyrus and the middle cingulate gyrus. The second putative **motor/somatosensory** network includes only the pre- and post-cruciate gyrus adjacent to the inter-hemispheric fissure and the rostral endo-marginal, marginal and ecto-marginal gyrus. A third putative **somatosensory** network only includes the rostral splenial gyrus, parts of the marginal and ecto-marginal gyrus.

One sample t-test maps of the identified RSNs, thresholded at P<0.05 are shown in S3 Fig.

## Discussion

This study proves the feasibility of rs-fMRI in anesthetized healthy beagle dogs and documents several brain RSNs, such as DMN, primary and higher order visual, auditory, somatosensory and sensory-motor.

In the past fMRI in dogs has been used mainly in specifically trained awake, healthy dogs to investigate aspects of canine cognition [43]. This approach requires months of training for each dog and is not feasible in a clinical setting. A further possible disadvantage of scanning dogs awake is movement during scanning and less motion was found in anaesthetised dogs compared to well-trained awake dogs in one study [16]. Performing fMRI under general anaesthesia therefore has clear benefits in dogs, but the influence of anaesthesia must be considered when interpreting the data. Before implementing rs-fMRI in a clinical setting, the presence of not only DMN, but also other networks has to be proven and reliably recognised under standardised anaesthesia regime, including standardised patient monitoring as it is routinely used for our daily clinical patients. Based on knowledge from literature, the standardised anaesthesia protocol was adjusted to minimise influence on the fMRI signal [44].

FMRI sequences were performed after the standard anatomical sequences, approximately one hour after induction of anaesthesia, allowing steady-state sevoflurane inhalation anaesthesia and minimising the influence of the medication used for induction. Anaesthetic depth was titrated to the minimum level needed for immobilisation and mechanical ventilation of the patient. This required a sevoflurane MAC between 0.8 to 1.2. The influence of anaesthesia on RSN has been investigate in humans [26], monkeys [27] and in rodents [28]. Not all networks are equally affected by anaesthetic agents. Higher-order networks linked to cognition are more severely affected than lower-order, such as basic sensorimotor networks [28]. Every anaesthetic drug uniquely modulates resting state connectivity [28]. Sevoflurane for example is associated with selectively reduced functional connectivity within cortical networks associated with consciousness [29]. Little is known about the influence of anaesthesia on RSN in dogs. The only study investigating the DMN in dogs found no significant differences DMN in dogs anaesthetised with Ketamine/Xylazine and awake dogs [16], but significantly greater connectivity of the posterior cingulate seed with voxels in the posterior regions of the brain was found in anaesthetised compared to awake dogs [16]. Therefore, comparing results from anaesthetised animals with those of awake ones bears the risk of anaesthesia related confounders. All these findings highlight the need of performing highly standardised and uniform studies either awake or under the same anaesthetic conditions, which enables to compare results between different groups. It is beyond the scope of this paper to discuss the best anaesthetic protocol for performing rs-fMRI. We have consequently used a standard protocol for this study, which is also applied for routine MRI examinations of clinical patients. This enables further investigation of RSN alterations in different canine diseases such as idiopathic epilepsy, cognitive dysfunction and behavioural diseases in the future. General anaesthesia was necessary for this study as clinical conditions do not allow training patients for awake and unrestrained fMRI examination.

The influence of changes in partial pressure of carbon dioxide and partial pressure of oxygen levels under sevoflurane anaesthesia on the fMRI signal has been shown in dogs [45]. To limit these influence EtCO2 was kept in physiological range between 35–38 mm/Hg and FiO2 between 55–65%.

Blood pressure was maintained within a physiological range in all patients as well as sudden changes were avoided to preclude influence of blood-pressure changes on fMRI signal [46].

Due to the ability of acquiring data from animals and humans in the same manner, rs-fMRI has emerged as a translational method for bridging experimental animal and human studies. In conjunction with data-driven gICA analysis, rs-fMRI has proven to be a robust approach to identify and characterise similar brain networks across species [5, 8, 9, 16, 47, 48].

Little is known about RSNs in dogs and for this reason a data driven approach was used to identify the RSN in our study. While gICA needs little assumption beforehand and results are easily reproducible within and in-between subjects, even using different number of components [12], one of the challenges is the correct labelling of the RSN. They can be labelled by macroscopic anatomy, cyto-architecture or function [49]. Stereotactic atlases exist for the human brain allowing localisation of the peak coordinates within a standardised space. These atlases are providing anatomical and cyto-architectonic labels, which give conclusion about the function of this area. Although stereotactic atlases exist in dogs, no functional correlation for these coordinates exists [50]. To overcome this issue, we used cross anatomy to identify functional areas previously localised in dogs in rs-fMRI and task-fMRI. This study compared the presented results with previously published RSNs in humans and other animals, for those canine functional areas without a reported correlate in fMRI. To enable cross anatomical comparison, results were registered to a high quality and study-specific T1 template that allows identification of anatomical structures easily.

We report the presence of seven RSN in the anaesthetised dogs similar to those previously shown in human and other animal data.

The DMN is involved in states of self-reference and has been extensively characterised in several other animals and humans. It is of particular interest due to its involvement and its relevance to mental disorders [51, 52]. In dogs DMN has been identified in anaesthetised and awake dogs and was found to be dissociated in an anterior and posterior component [16]. In the above-mentioned study, results were relatively consistent in all dogs, only minor differences were found between both groups with increased connectivity in the anaesthetised versus awake dogs. Highlighting reproducibility of rs-fMRI, the two components of DMN were also clearly identified in the present study, although different anaesthetic protocol and the different number of IC were chosen. Recently, anterior and posterior components of DMN were described in ferrets by means of ICA as well [8].

Weaker connectivity between the anterior and posterior component in dogs has been supported using diffusion tensor tractography, which only identified white matter tracts connecting medial prefrontal cortex/anterior cingulate cortex to the posterior cingulate cortex in 9/23 dogs [53], while in humans these tracts were identified in 22/23 subjects [54]. This was also reflected by a significantly lower group fractional anisotropy in dogs in this area compared to humans [53]. Dissociation of the DMN has been found in children and in patients with Alzheimer`s diseases and may reflect a lower level cognitive processing [52]. Strikingly, the components of the DMN could not be clearly identified in the study of Szabó et al. [17]. In contrast to our study anterior-posterior connectedness was found, but the component included also areas not included in the human DMN [17].

Analogy between canine and human visual and auditory cortex functions have been shown by previous task-fMRI. Therefore, it was expected to find correspondence also in the RSNs.

We found two visual networks reflecting primary and higher-order separations seen in the human and animal studies, including awake dogs [9, 17, 55]. Furthermore, this primary visual network was in consistency with results from task-fMRI in awake and anaesthetised healthy dogs after visual stimulation [15, 37]. In these studies, a second response was also noticed reflecting higher order visual networks (for face processing, only on the right side and suspected motion perception, on both sides) overlapping with the higher order visual network identified in our study.

The auditory network identified in our study corresponds with the primary auditory cortex in dogs and auditory RSN identified in awake dogs [17]. Activation of this area by sound has been shown in previous studies in awake unrestrained dogs [14, 56]. While Andics and colleagues showed common functions in dog and human voice processing [14], Prichard et al. reported that most dogs were able to discriminated pseudo-words from trained words [56]. Interestingly, in anaesthetised dogs, Bach and colleagues found only subcortical but no cortical response to auditory stimuli [36]. The authors discuss the possibility of anaesthesia precluding activation of cortical areas. The effect of anaesthesia on fMRI signal is well known and cortical response to auditory stimuli has been identified in anaesthetised cats [57] and rats [58] using different anaesthesia protocols.

RSNs found in the parietal and temporal cortex were analysed in the context of previous human studies and this finding should be therefore carefully interpreted. While the primary motor and sensory cortex is well defined in dogs [59], precise organisation of higher order networks is not well known. Two of the these RSN (networks 6 and 7) include primary motor cortex areas in the pre- and post-cruciate gyrus and the primary sensory cortex areas in the post-cruciate and supra-sylvian gyrus. These two RSN appear similar to the RSN labelled “Primary sensorimotoric, premotoric and supplementer motoric regions” and “Primary and associative sensory cortical areas” by Szabó and colleagues [17].

Regarding network 5, neither primary motor nor primary sensory cortex areas are included and for this reason the function of this network can only be speculated. This is similar to a corresponding RSN in humans in the superior parietal lobule which was associated with action and somesthesis [60]. This network is overlapping with the RSN labeled “External limbic circle (mid cingulate cortex)” by Szabó and colleagues in their IC_15_ [17].

**Fig 3:**
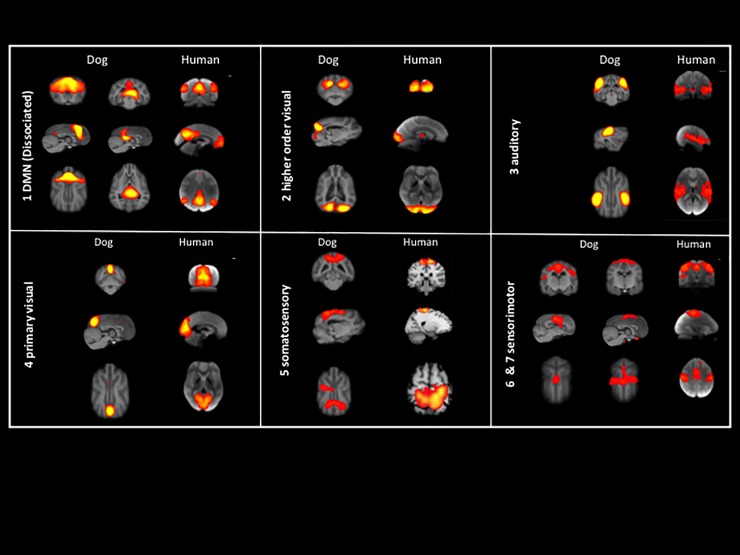
Comparison of generated RSNs of the present study with published RSNs in humans, both registered to T1W anatomical templates. RSN 1: DMN; RSN 2: higher order visual; RSN 3: auditory; RSN 4: primary visual; RSN 5: somatosensory; RSN 6 and 7: sensorimotor. Images from human RSNs adapted from Smith et al., 2009 (RSNs 1–4 and 6–7) and Laird et al., 2013 (RSN 5).

In summary, RSN in dogs are anatomically similar to networks found in humans (Fig 3) and other animals [5, 8, 9, 47, 48, 61]. Our results are in general agreement with the recent rs-fMRI study in awake dogs, with 6 out of 7 of our RSNs matching in both studies [17]. Only one RSN, the DMN, does not match in both studies. While our results correspond to the results of Kyathanahally et al. [16], that showed dissociated DMN in dogs in rs-fMRI as well as in DTI [53], this could not be reproduced by Szabó and colleagues [17].

Fig 3 illustrates similarities by comparing our results to RSNs previously published in humans [12, 60].

An important limitation of the present study is the lack of corresponding electrophysiological examinations of the brain. The RSNs have been positively correlated with alpha activity seen in electroencepaholography in humans [62, 63]. Further research, including simultaneous electroencephalographic recordings, would be helpful to better define the functional cortical organisation of the canine brain and to characterise these networks.

Furthermore, no direct comparison has been performed with awake dogs or dogs under a different anaesthetic protocols to demonstrate the influence of anaesthesia on detection of RSN.

Even if task-fMRI has been performed in anaesthesia before and results match to RSN in our study, a direct intra-individual comparison is missing. Performing task-fMRI under the current conditions would have supported the resting state results and supported the choice of anaesthesia protocol.

Identifying RSN in dogs under standardised general anaesthesia opens the door to investigate pathophysiological changes in brain diseases under clinical settings. This may play a major role when no structural abnormalities are found during examination. Since MRI as a non-invasive technique, is routinely performed in dogs with brain disease, it allows to investigate naturally occurring diseases as translational large animal model.

## Conclusion

Using ICA method, we identified 7 RSNs common to those of awake dogs and to other mammals including humans. This study sets a basis for investigating pathophysiological characteristics of various canine brain diseases.

## Supporting information

S1 Fig Pre-processing of the anatomical images. This figure shows how the raw-data were first converted into a FMRIB Software Library v6.0 compatible format and then further pre-processed using this software.
(TIFF)

S2 Fig Artefactual components from the 20-component ICA decomposition of the resting fMRI data. The RS components do not lie within grey matter and are caused most likely by confound factors such as variations in subjects’ head sizes, head motion, and non-neural physiological fluctuations.
(TIFF)

S3 Fig One sample t-test maps of the anterior and posterior DMN, the auditory network, the primary and higher order visual network, the somatosensory, and the two putative sensory motor networks identified. Maps are thresholded at p<0.05.
(TIFF)
