# Supplementary figures and images for: Resting state networks of the canine brain under sevoflurane anaesthesia
Source: PLoS One. 2020 Apr 17;15(4):e0231955. doi: 10.1371/journal.pone.0231955 (PMC7164650; doi:10.1371/journal.pone.0231955)

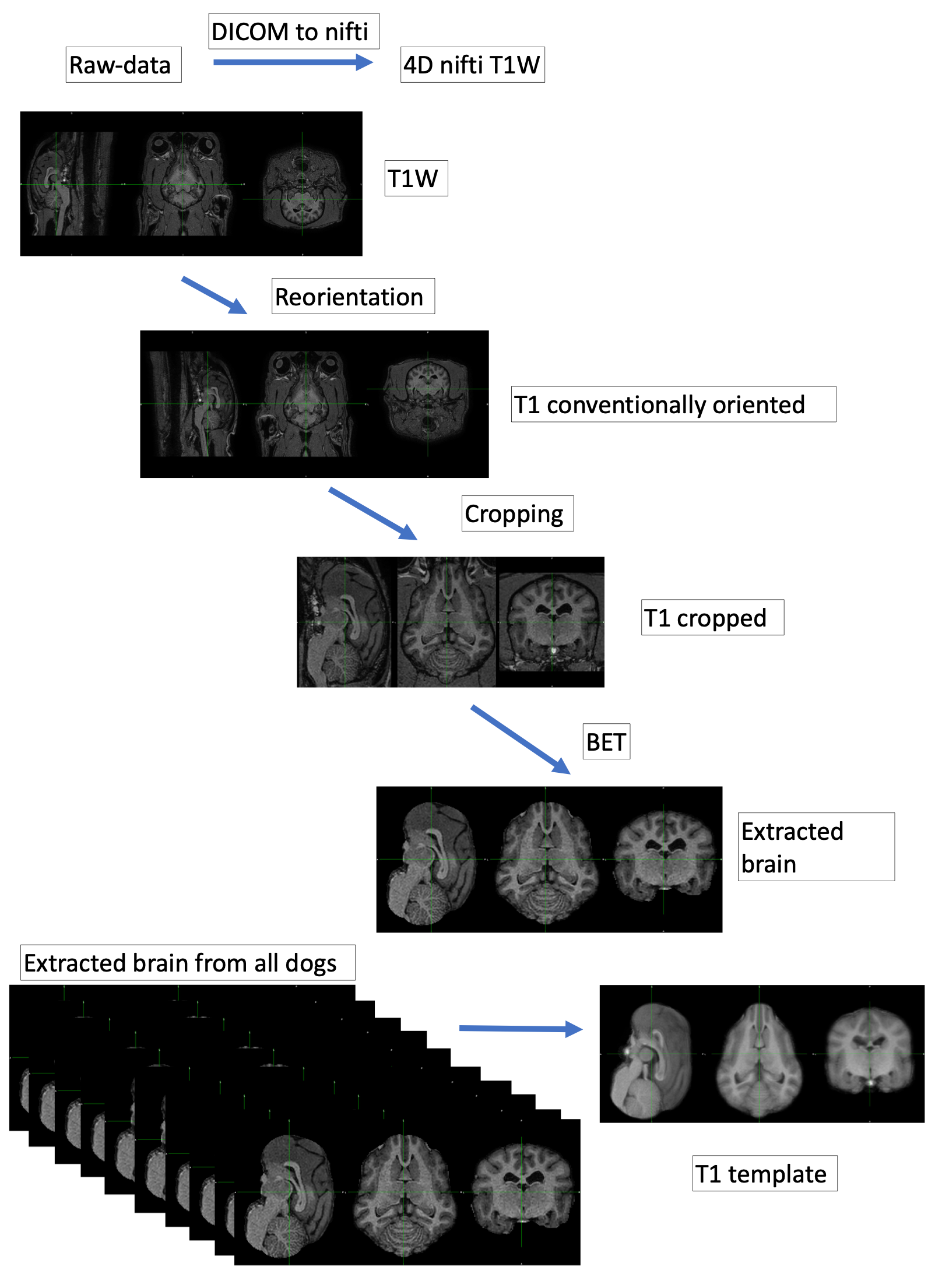

Supplement: S1 Fig — This figure shows how the raw-data were first converted into a FMRIB Software Library v6.0 compatible format and then further pre-processed using this software. (TIFF) [file pone.0231955.s001.tiff]

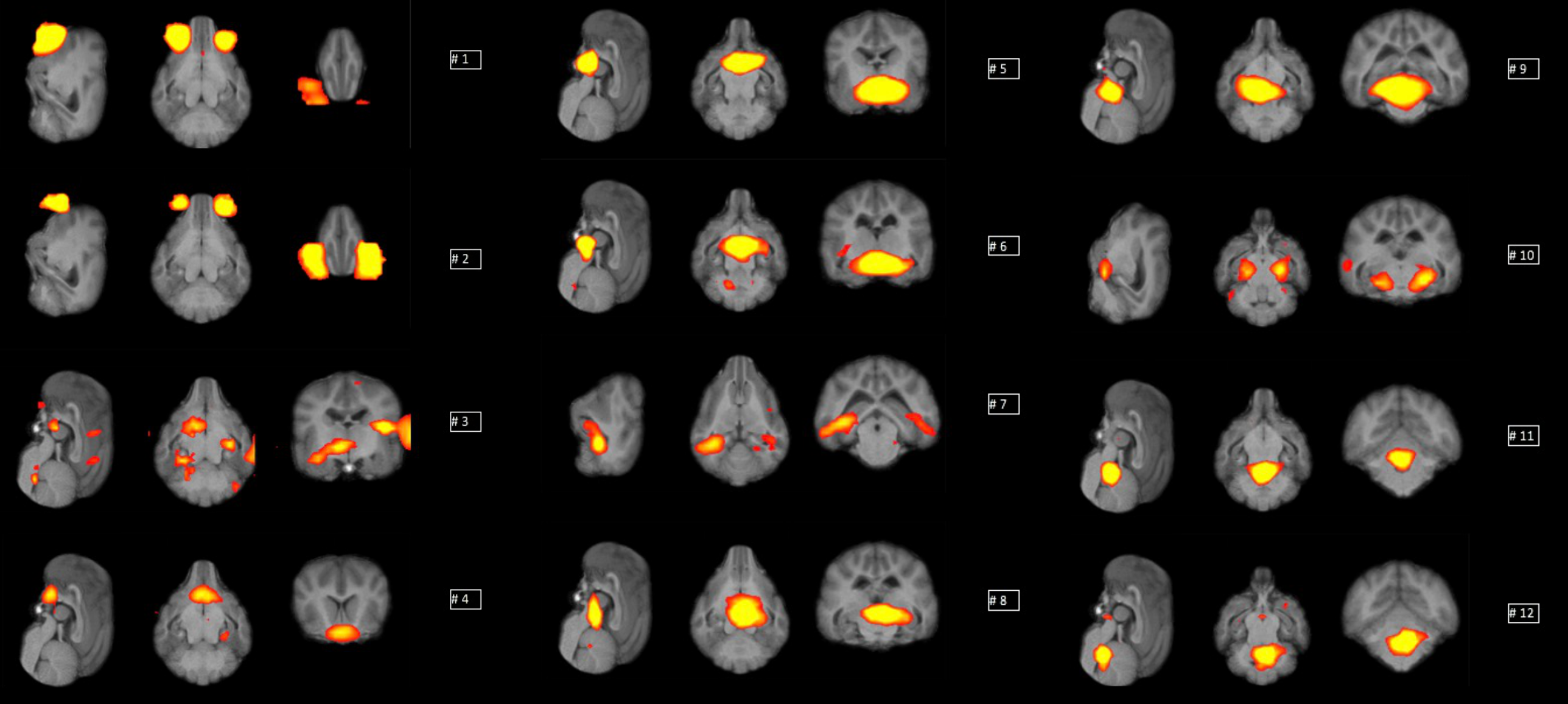

Supplement: S2 Fig — The RS components do not lie within grey matter and are caused most likely by confound factors such as variations in subjects’ head sizes, head motion, and non-neural physiological fluctuations. (TIFF) [file pone.0231955.s002.tiff]

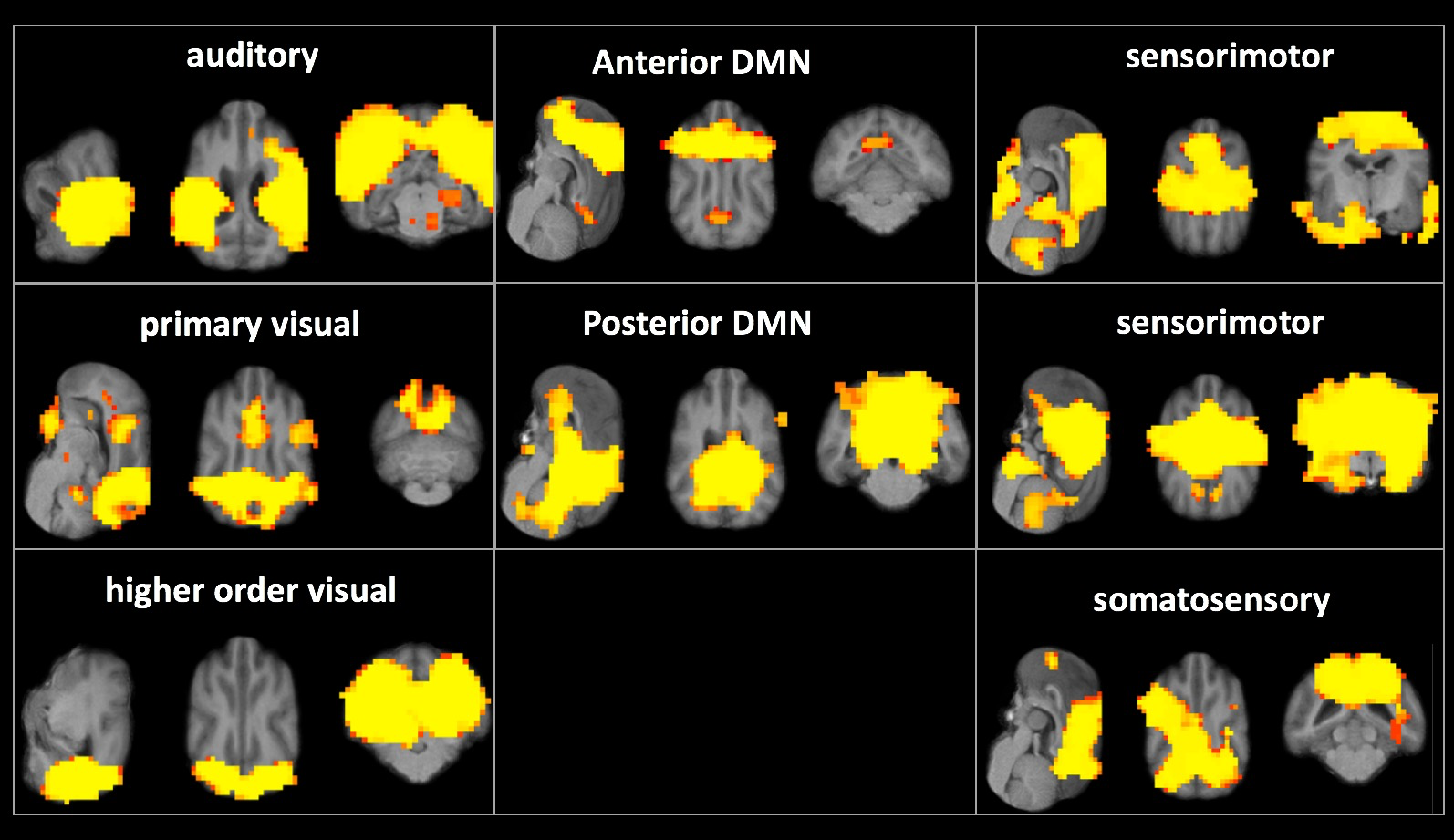

Supplement: S3 Fig — Maps are thresholded at p<0.05. (TIFF) [file pone.0231955.s003.tiff]
